# The Predictive and Prognostic Significance of c-erb-B2, EGFR, PTEN, mTOR, PI3K, p27, and ERCC1 Expression in Hepatocellular Carcinoma

**DOI:** 10.5812/hepatmon.7492

**Published:** 2012-10-11

**Authors:** Nuray Bassullu, Ilknur Turkmen, Murat Dayangac, Pinar Yagiz Korkmaz, Reyhan Yasar, Murat Akyildiz, Onur Yaprak, Yaman Tokat, Yildiray Yuzer, Gulen Bulbul Dogusoy

**Affiliations:** 1 Department of Pathology, Istanbul Bilim University Medical Faculty, Istanbul, Turkey; 2 Department of General Surgery, Florence Nigthingale Hospital, Istanbul, Turkey; 3 Department of Pathology, Florence Nigthingale Hospital, Istanbul, Turkey; 4 Department of Gastroenterology, Istanbul Bilim University Medical Faculty, Istanbul, Turkey; 5 Department of General Surgery, Florence Nigthingale Hospital, Istanbul, Turkey

**Keywords:** Carcinoma, Hepatocellular, Immunohistochemistry

## Abstract

**Background:**

Hepatocellular carcinoma (HCC) is the fifth most common fatal cancer and an important healthcare problem worldwide. There are many studies describing the prognostic and predictive effects of epidermal growth factor receptor 2 (c-erb-B2) and epidermal growth factor receptor 1 (EGFR), transmembrane tyrosine kinases that influence cell growth and proliferation in many tumors.

**Objectives:**

The current study aimed to investigate the expression levels of c-erb-B2, EGFR, PTEN, mTOR, PI3K, p27, and ERCC1 in hepatocellular carcinoma (HCC) and their correlation with other clinicopathologic features.

**Patients and Methods:**

Fifty HCC cases were stained immunohistochemically with these markers. Correlations between the markers and clinicopathologic characteristics and survival rates were analyzed.

**Results:**

No membranous c-erb-B2 staining was seen, whereas cytoplasmic positivity was present in 92% of HCC samples, membranous EGFR was observed in 40%, PI3K was found in all samples, and mTOR was seen in 30%, whereas reduced or absent PTEN expression was observed in 56% of samples and loss of p27 was seen in 92% of the cases. c-erb-B2 and mTOR overexpression, as well as reduced expression of p27, all correlated with multiple tumors (P = 0.041, P < 0.001, and P < 0.001, respectively). P27 loss, and mTOR and EGFR positivity were significantly correlated with AFP (P = 0.047, P = 0.004, and P = 0.008, respectively). Angiolymphatic invasion was more commonly seen in EGFR- and ERCC1-positive cases (P = 0.003 and P = 0.005). EGFR was also correlated with histological grade (P = 0.039). No significant correlations were found among PTEN , PI3K, and the clinicopathological parameters. Disease-free or overall survival rates showed significant differences among therapy modalities, AFP levels, angiolymphatic or lymph node invasions, and ERCC1 and p27 expression levels (P < 0.05).

**Conclusions:**

c-erb-B2, EGFR, mTOR, ERCC1 overexpression levels, and loss of p27 may play roles in hepatocarcinogenesis and may be significant predictors of aggressive tumor behavior. These markers were found to be correlated with certain clinicopathologic features, therapy modalities, and survival rates in the current study. These findings may help in planning new, targeted treatment strategies .

## 1. Background

Hepatocellular carcinoma (HCC) is the fifth most common fatal cancer and an important healthcare problem worldwide, especially in Asia, where its incidence is increasing in many countries. Despite advances in clinical research, HCC prognosis remains poor and is currently the third most common cause of cancer death worldwide (1-3). HCC carcinogenesis is a multistep process with many possible etiologic risk factors, including hepatitis B and C viruses (HBV and HCV), aflatoxin exposure, chronic alcohol consumption, nonalcoholic steatohepatitis, α1-antitrypsin deficiency, cigarette smoking, and elevated endogenous testosterone in serum (4, 5). Conventionally, HCC prognosis has been primarily based on tumor stage and histologic grade (6, 7) in addition, angiolymphatic invasion (ALI) and high alpha fetoprotein (AFP) levels are known to correlate with shorter disease-free survival (DFS) (7). The prognosis of HCC patients remains poor, and useful prognostic and predictive molecular markers are required. Some recent molecular factors such as tumor proliferative indices, nuclear DNA ploidy, and levels of growth factors and hormone receptors have been used to predict clinical outcome in HCC patients (6). There are many studies describing the prognostic and predictive effects of epidermal growth factor receptor 2 (c-erb-B2) and epidermal growth factor receptor 1 (EGFR), transmembrane tyrosine kinases that influence cell growth and proliferation in many tumors (8). However, controversial results have been reported for HCC (6, 7, 9-18). Immunoreactivity of anti-c-erb-B2 antibodies has ranged from 0% (6, 13) to 92.3% (12) in different studies. Although there are some reports of c-erb-B2 overexpression or amplification in HCC (17, 18), other authors found that neither overexpression nor amplification was seen in HCC, hepatocellular adenoma, or normal liver tissue. (6, 13). EGFR levels also vary; overexpression has been observed between 4.2% and 85% of HCCs in previous studies (7, 11-13).

Signaling pathways involving phosphatase and tensin homolog deleted on chromosome ten (PTEN), mammalian target of rapamycin (mTOR), phosphatidylinositol 3’-kinase (PI3K) also regulate cell proliferation and survival and have been investigated in several studies of carcinogenesis (19-24). PTEN, a tumor suppressor gene, is a negative regulator of PI3K-Akt signaling. Immunohistochemically, PTEN loss is correlated with mTOR overexpression (22). Villanueva et al. (24) reported that aberrant mTOR signaling was present in 50% of HCC cases. During progression through the cell cycle, the cyclin-dependent kinase inhibitor p27 negatively regulates the G1 phase. Decreased p27 expression is closely associated with poor prognosis in cases of HCC. Low p27 immunoreactivity is also significantly correlated with tumor invasiveness, advanced clinical stage, and poor cellular differentiation (25-32). Excision repair cross-complementation group 1 (ERCC1) is a key DNA repair enzyme (33-35). Fautrel et al. (33) reported that increased ERCC1 expression is associated with liver fibrogenesis and cancer.

## 2. Objectives

The current study aimed to investigate the expression levels of c-erb-B2, EGFR, PTEN, mTOR, PI3K, p27, and ERCC1 in HCC cases by conventional immunohistochemistry (IHC) methods and assessed correlations among staining intensities, clinicopathologic features, and survival rates in 50 patients who underwent liver resections to treat HCC.

## 3. Patients and Methods

### 3.1. Patients

All 50 patients with HCC had total hepatectomy and transplantation, except two cases that had partial hepatectomy. Clinicopathological data including age, gender, etiological factor, tumor size, number, level of AFP, and treatment modality were retrieved from surgery archives and pathology reports. None of the patients had received neoadjuvant treatment. Child Pugh and model for End-stage Liver Disease (MELD) scores and Milan’s criteria for transplantation were evaluated (36, 37). Histological grade was assessed as well-differentiated (grade 1), moderately differentiated (grade 2), poorly differentiated (grade 3), and undifferentiated (grade 4) according to the modified Edmondson and Steiner criteria and staged according to the TNM classification of malignant tumors, 7th edition (38).

### 3.2. Histopathological Evaluation

Tissue samples were processed immediately after surgical removal. For histologic examination, all specimens were fixed in 10% of neutral buffered formalin and embedded in paraffin after overnight tissue processing. Standard 4-μm tissue sections were prepared and stained with hematoxylin and eosin for light microscopy examination.

### 3.3. Immunohistochemistry

IHC was performed on 4-μm sections comprised of tumorous and nontumorous tissues. Staining was done manually by a standard streptavidin-biotin-peroxidase method. Paraffin-embedded tissue sections were baked in a 60°C oven overnight, deparaffinized through three changes of xylene, and rehydrated through a series of decreasing concentrations of ethanol solutions to distilled water. After deparaffinization and rehydration, antigen retrieval was performed by microwave cooking at 600W for 20 minutes in 10 mM citrate buffer, pH 6.0, and then left to cool at room temperature for 20 minutes. Endogenous peroxidase activity was quenched in 3% hydrogen peroxide in methanol for 5 minutes at room temperature and washed in phosphate-buffered solution (PBS) for 10 minutes. After blocking nonspecific antibody binding with ultraviolet block for 10 minutes, tissue sections were incubated with monoclonal antibodies against c-erb-B2, EGFR, p27, PTEN, MTOR, PI3K, and ERCC1, and positive controls were included according to the manufacturers’ recommendations (Table 1). AEC (3-amino-9-ethylcarbazole) and DAB (3, 3’ diaminobenzidine) were used as chromogens. Sections were rinsed in water, counterstained with hematoxylin, and mounted with cover-slips.

**Table 1:** IHC Antibodies

Antibody	Clone	Species	Dilution	Source	Positive Control
**c-erb-B2**	CB11	Mouse monoclonal	RTU	Novocastra	Breast carcinoma
**EGFR**	EGFR.113	Mouse monoclonal	1/20	Novocastra	Placenta
**P27**	1B4 NCL-P27	Mouse monoclonal	1/30	Novocastra	Tonsil
**PTEN**	28H6 GTX73862	Mouse monoclonal	1/60	Genetex	Prostate
**MTOR**	EPR426	Rabbit monoclonal	1/100	Genetex	Breast carcinoma
**PIK3C**	-	Rabbit polyclonal	1/70	Abnova	Breast carcinoma
**ERCC1**	SP68	Rabbit monoclonal	1/100	Spring	Placenta

### 3.4. IHC Evaluation

The evaluation of c-erb-B2 immunoreactivity was performed according to DAKO (Denmark) protocol for the Hercept Test, with minor modifications. Usually only membrane staining is considered positive. As membranous staining was not observed in the cases under study, cytoplasmic staining was considered both in tumors and surrounding cirrhotic liver tissues and was evaluated as present or absent. If present, intensity was scored as weak, moderate, or strong (16, 17). For EGFR, membranous staining intensity was scored in four categories: no staining (0); weak staining (1+);( light brown membrane staining, visible only with high power magnification), intermediate staining (2+, between 1+ and 3+); and strong staining (3+, visible with low power magnification, dark brown staining delineating the membrane). The following formula was employed to integrate the data relating to staining intensity and frequency to calculate IHC scores: 1 × (percentage of weakly stained cells [1 +]) + 2 × (percentage of moderately stained cells [2 +]) + 3 × (percentage of strongly stained cells [3 +]) (39). Nuclear immunoreactivity for p27 was considered positive when > 40% of the tumor cells were stained. Nuclear immunoreactivity for ERCC1 was accepted as positive. Slide was scored as 0 if 0% of the tumor cells were positive, 0.1 if 1-9%, 0.5 if 10-49%, and 1.0 if 50% or more were positive. A semiquantitative H-score was calculated by multiplying the staining intensity (0-3) by the percentage score. The tumor was considered positive when the H-score was > 1.0 (40). In the immunohistochemical evaluation of PTEN and MTOR; the percentage of positive cells was scored as follows: 0, no staining or staining in < 5% of the tumor cells; 1, staining in 5-25% of cells; 2, staining in 26-50% of cells; 3, staining in 51-75% of cells; and 4, staining in > 75% of cells. Staining intensity was scored as 0 (negative), 1 (weak), 2 (moderate), or 3 (strong). Next, immunoreactivity score (IRS) was calculated by multiplying the percentage and the staining intensity score. Results of 0-6 were considered negative, and values of 7-12 were considered positive (41).

### 3.5. Statistical Analysis

SPSS 8.0 was employed to analyze IHC scoring data. Relationships were analyzed using Fisher’s exact and Pearson’s (chi-squared) tests. Kaplan–Meier survival curves and log-rank tests were used for survival analysis. Values were considered statistically significant at P < 0.05.

## 4. Results

### 4.1. Clinical Characteristics

The patients’ mean age was 56.82 years, and the median age was 57. Ages ranged from 26-72, and the male/female ratio was 44/6. Ninety percent (45/50) of patients were older than 50 years. It was determined that 31 cases (62%) were positive for HBV, 6 cases were positive for HCV, 3 cases had both HBV and HCV, 2 cases had HBV and Hepatitis D virus (HDV), 5 cases had Laennec’s cirrhosis, and 3 cases showed evidence of cryptogenic cirrhosis. Regarding the cases with Child Pugh Score, 27 cases were classified as A, 16 cases as B, and 7 as C. Among 48 total hepatectomy cases, 24 cases had MELD score ≤ 10, 20 cases were 11-19, and 3 cases ≥ 20. Twenty-five met Milan’s criteria, and 23 did not. Serum AFP levels were available for 47 patients. It was higher than 25 μg/L in 27 patients and lower than 25 μg/L in 20 patients.

### 4.2. Pathological Characteristics

**Figure 1:**
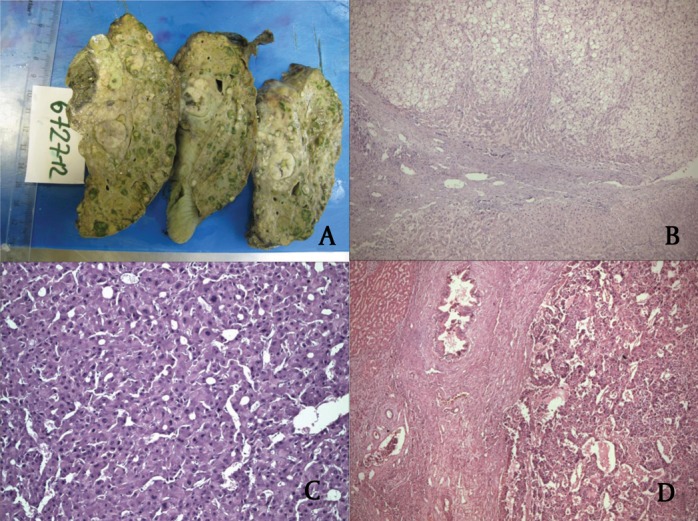
Macroscopic and Microscopic Views of Hepatocellular Carcinoma A) Multifocal HCC. B) Grade I. C) Grade II. D) Grade III Hepatocellular Carcinoma (HE; × 100, × 100, × 200)

The size of the largest tumor nodule was considered as tumor size. The mean size was 4.2 cm (range: 1-14 cm). Seven tumors were smaller than 2 cm, 16 were 2.1-3 cm, 17 were 3.1-4.9 cm, and 10 were larger than 5 cm. Among the 50 cases, 18 and 32 had solitary and multiple tumors, respectively (Figure 1A). Of those with multiple tumors, 11 cases had two, 21 cases had more than three, and one exceptional case had 20 tumor nodules. Nineteen patients had bilobar tumors, and 31 cases had tumors limited to the right lobe. Liver cirrhosis was grossly and microscopically present in all HCC cases. Necrosis was observed in 15 cases, and ALI was present in nine cases. Lymph node metastasis and tumor thrombi in the portal vein were detected in one and two cases, respectively. Histological grading according to modified Edmondson and Steiner criteria (38) revealed that six cases were grade 1 (12%), 35 cases were grade 2 (70%), and the nine remaining cases were grade 3 (18%) (Figure 1B-D). Based on the international TNM pathological staging criteria (38), three cases were stage I (6%), 19 cases were stage II (38%), 12 cases were stage III (24% ) and 16 cases were Stage IV (32%).

### 4.3. Treatment and Follow-up

The average follow-up period was 38.7 months (range: 2-87 months). Forty patients survived more than 2 years, and 10 died within 2 years. The clinicopathological variables are listed in Table 2. None of the patients received neoadjuvant treatment. Thirty-four patients received a calcineurin inhibitor (CNI, cyclosporine or tacrolimus), eight received mTOR inhibitor therapy (MIT, sirolimus or everolimus), and eight did not receive postoperative treatment (NT).

**Table 2:** Correlations of Clinicopathological Characteristics and IHC Results in HCC a

	C-erb-B2, No. (%)	EGFR, No. (%)	PTEN, No. (%)	MTOR, No. (%)	PI3K, No. (%)	P27, No. (%)	ERCC1 H > 1, No. (%)	*P *value
**Age**								NS
< 50	5 (10)	3 (6)	2 (4)	2 (4)	5 (10)	0 (0)	0 (0)	
> 50	41(82)	17(34)	20 (40)	13 (26)	45 (90)	4 (8)	10 (38.4)	
**Gender**								NS
Female	41(82)	18 (36)	19 (38)	13 (26)	44 (88)	0 (0)	9 (34.6)	
Male	5 (10)	2 (4)	3 (6)	2 (4)	6 (12)	4 (8)	1 (3.8)	
**Tumour Size **								NS
≤ 2 cm	6 (12)	3 (6)	1 (2)	3 (6)	7 (14)	0 (0)	0 (0)	
2.1-3 cm	15 (30)	7 (14)	8 (16)	4 (8)	16 (32)	1 (2)	4 (15.3)	
3.1-5 cm	15 (30)	7 (14)	8 (16)	7 (14)	17 (34)	2 (4)	6 (23)	
≥ 5 cm	10 (20)	3 (6)	5 (10)	1 (2)	10(20)	1 (2)	0 (0)	
**Multiplicity**								with c-erb- B2 *P* = 0.041
								with p27 *P* < 0.001
								With mTOR *P* < 0.001
Single	18 (36)	9 (18)	11 (22)	5 (10)	18 (36)	1 (2)	2 (7.7)	
Multiple	28 (56)	11 (22)	11 (22)	10 (20)	32 (64)	3 (6)	8 (30,7)	
**Etiology**								NS
HBV	29 (58)	11	15 (30)	7 (14)	31	4 (8)	6 (23.07)	
HCV	6 (12)	2 (4)	2(4)	3 (6)	6	0 (0)	2 (7.7)	
HBV HCV	2 (4)	2 (4)	2 (4)	1 (2)	3 (6)	0 (0)	1 (3.8)	
HBV HDV	1 (2)	1 (2)	1 (2)	0 (0)	2 (4)	0 (0)	0 (0)	
Alcohol	5 (10)	3 (6)	1 (2)	2 (4)	5	0 (0)	1 (3.8)	
Cryptogenic	3 (6)	1 (2)	1 (2)	2 (4)	3 (6)	0 (0)	0 (0)	
**ALI**								with EGFR *P* = 0.0031
								with ERCC *P* = 0.005
Yes	8 (16)	4 (8)	4 (8)	1 (2)	9 (18)	2 (4)	3 (11.5)	
No	38 (72)	16 (32)	18 (36)	14 (28)	41 (82)	2 (4)	7 (26.9)	
**PVT**								NS
Yes	2 (4)	1 (2)	1 (2)	0 (0)	2 (4)	0 (0)	1 (3.8)	
No	44	19 (38)	22 (44)	15 (30)	0 (0)	4 (8)	9 (34.6)	
**LN Metastasis**								NS
Yes	1 (2)	0 (0)	0 (0)	0 (0)	1 (2)	1 (2)	0 (0)	
No	45 (90)	20 (40)	22 (44)	15 (30)	49 (98)	3 (6)	10 (38.4)	
**Grade **								with EGFR *P* = 0.039
I	5 (10)	1 (2)	2 (4)	3 (6)	6 (12)	0 (0)	2 (7.7)	
II	32 64)	17 (34)	14 (28)	9 (18)	35 (70)	1 (2)	5 (19.2)	
III	9 (18)	2 (4)	6 (12)	3 (6)	9 (18)	3 (6)	3 (11.5)	
**Stage**								NS
I	3 (6)	1 (2)	1 (2)	1 (2)	3 (6)	0 (0)	0 (0)	
II	18 (36)	10 (20)	11(22)	5 (10)	19 (38)	1 (2)	3 (11.5)	
III	11 (22)	3 (6)	4 (8)	4 (8)	12 (24)	1 (2)	3 (11.5)	
IV	14 (28)	6 (12)	6 (12)	5 (10)	16(32)	2 (4)	4 (15.3)	
**AFP**								with P27 *P* = 0,047
								with EGFR *P* = 0,008
								with mTOR *P* = 0,04
≤ 25	17 (34)	20 (40)	6 (12)	6 (12)	20 (40)	1 (2)	5 (19.2)	
> 25	29 (58)	0 (0)	16 (32)	9 (18)	30 (60)	3 (6)	5 (19.2)	
Total Positive Cases	46 (92)	20 (40)	22 (44)	15 (30)	50 (100)	4 (8)	10 (38.4)	

Abbreviations: ALI, angiolymphatic invasion; LN, lymph node; NS, not significant; PVT, portal venous thrombus.

^a^n = 26 cases.

### 4.4. Statistical Analysis Results

**Figure 2:**
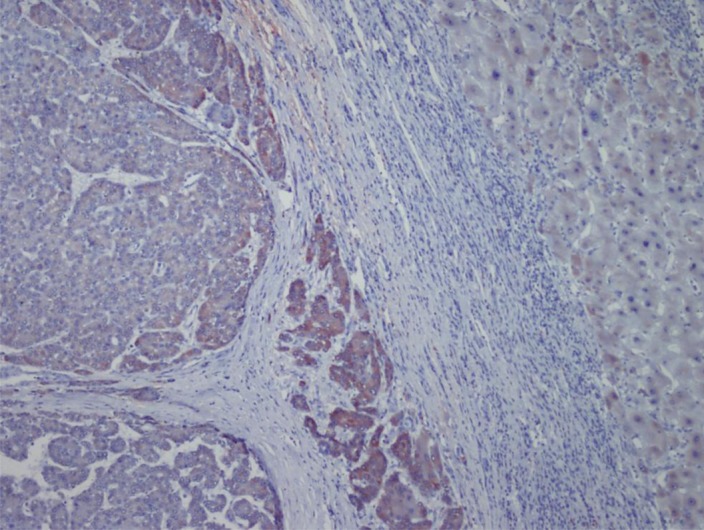
Cytoplasmic 3+ Staining of c-erb-B2 in HCC and 1+ Staining in Adjacent Liver Parenchyma (× 100)

**Figure 3:**
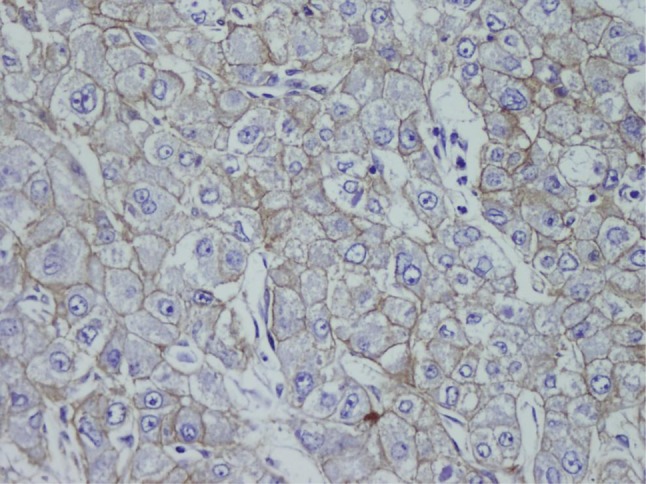
Membranous EGFR Staining (× 400)

**Figure 4:**
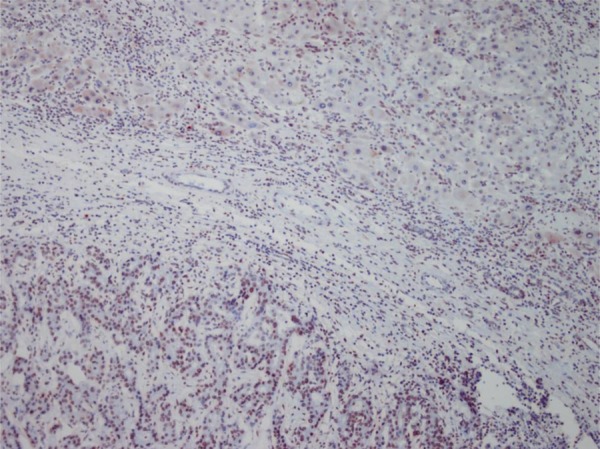
PTEN Showing Nuclear Positivity in HCC, While Noncancerous Tissue is Negative The positive Signal in the Paranchyma is Attributable to Lymphocytes (× 100)

**Figure 5:**
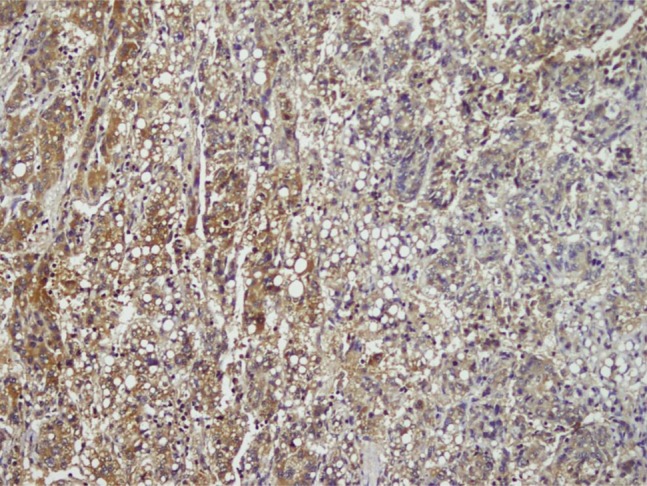
Moderate to Strong Cytoplasmic PI3K Staining (× 200)

**Figure 6:**
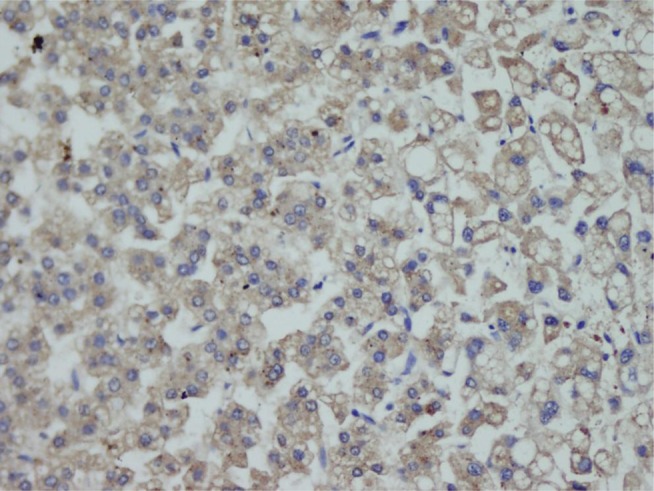
Moderate Cytoplasmic mTOR Staining (× 200)

**Figure 7:**
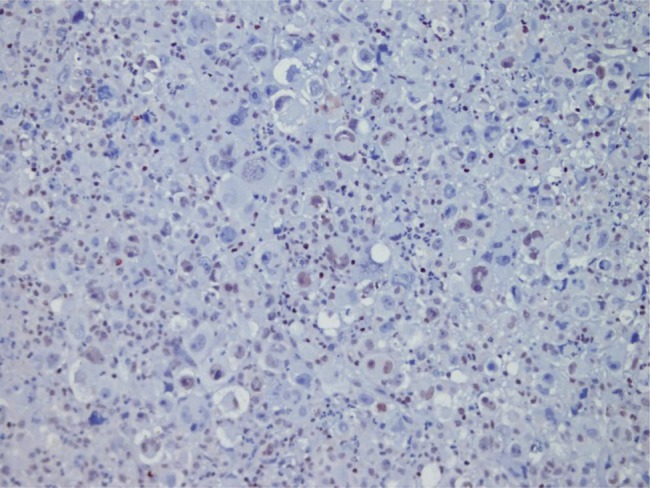
P27 Expression in HCC (× 200)

**Figure 8:**
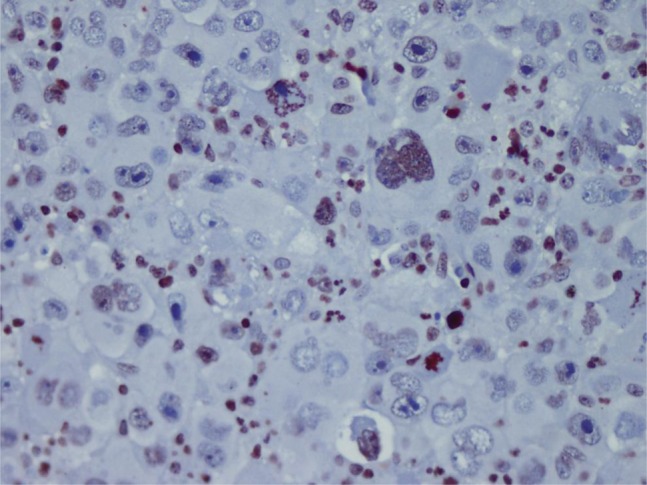
ERCC Nuclear Expression in HCC (× 400)

HBV was more common in patients with advanced cancer stages (P = 0.04) and right lobe tumors (p < 0.022), although all tumors in HCV (+) patients were larger (P < 0.001). Tumor size was significantly larger in patients older than 50 years (P = 0.04). Tumor diameter correlated with necrosis, AFP level, and multiplicity (P = 0.054, P < 0.001, and P = 0.021 respectively). Membranous c-erb-B2 staining was not observed in the cases under study. Cytoplasmic positivity was present in 92% (46 cases) (Figure 2). When compared with the pathologic parameters, correlations were only found for multiplicity (P = 0.04) and p27 expression (P = 0.01). EGFR membranous positivity was present in 40% of cases (Figure 3) and significantly correlated with grade (P = 0.039), AFP level (P = 0.008), and ALI (P = 0.003). PTEN expression was reduced or absent in 28 (56%) of patients (Score I: n = 18, II: n = 10, II: n = 7, IV: n = 15) (Figure 4). PI3K was moderately positive in all cases (Figure 5). No significant associations were found among PTEN, PI3K, and pathological parameters. MTOR was positive in 15 cases (Figure 6) and correlated with both AFP level (P = 0.04) and multiplicity (P < 0.001). P27 loss was observed in 92% of cases (Figure 7). AFP level was < 25 μg/L in one of the four p27-positive cases, which was statistically significant (P = 0.047). Notably, three of the four p27-positive cases had multiple tumors (P < 0.001). ERCC1 could be studied in 26 cases. Three of which were completely negative, and 13 cases had positive nuclear staining (Figure 8). The H score was determined to be < 1 and correlated with ALI (P < 0.005). When the survival analysis was performed according to the Kaplan–Meier curve and log-rank tests, differences between treatment regimens; presence of ALI; lymph node (LN) metastasis; and c-erb-B2, p27, and ERCC1 expression levels were found statistically significant. Patients who received CNIs showed a significantly longer DFS and overall survival (OS) when compared with MIT and NT patients (P < 0.001). The DFS and OS ranges were 74.4-75 months for the CNI group, 49.6-59 months for the MIT group, and 27.8-28 months for the NT group. The DFS and OS were 69.3 versus 17.7 months and 70 months versus 19 months in the absence and presence of ALI, respectively (P = 0.039 and 0.019, respectively). When LN metastasis was present, the average OS decreased from 67 to 9 months (P = 0.044). The difference of OS in cases with mild and intense positivity of c-erb-B2 (72.5 and 32.8 months respectively) was also significant (P = 0.012). Similarly, patients with an ERCC1 H-score < 1 had a significantly longer OS (66 months) than the group with H scores > 1 (25 months, P < 0.001). p27 expression also correlated with OS; the p27-negative group had an average OS of 69 months, and the p27-positive group only survived for an average of 7.7 months (p = 0.002).

## 5. Discussion

The demographic and clinicopathologic findings of the current series were in agreement with other studies (37, 42), as previously reported(43). The prognosis for HCC patients remains poor, and the identification of useful prognostic and predictive molecular markers may help improve DFS and OS. Some studies have shown the prognostic and predictive utility of markers such as c- erb-B2 and EGFR, which influence cell growth and proliferation, in many types of tumor. The c-erb-B2 (also known as HER-2/neu) proto-oncogene is a transmembrane growth factor receptor in the tyrosine kinase receptor family. Its overexpression and/or amplification is known to be correlated with neoplastic transformation and progression in breast cancer and is associated with other poor prognostic factors, including stage, grade, and resistance to drug treatment. It also has an established role in many carcinomas, such as lung, gynecological, and gastrointestinal tumors (8). However, controversial results have been reported for HCC (6, 7, 9-18). In previous studies, c-erb-B2 immunoreactivity has ranged from 0% (6, 13) to 92.3% (12). While there are some studies that found c-erb-B2 overexpression or amplification in HCC (17, 18), some authors were unable to support these findings in HCC, hepatocellular adenoma, or normal liver tissue (6, 13). Membranous expression (9, 10, 14, 15) and cytoplasmic expression (16, 17) have both been reported. Membranous c-erb-B2 staining was not observed ,but cytoplasmic positivity was present in 92% of the cases. Studies assessing c-erb-B2 expression in HCC have demonstrated correlations with portal cirrhosis, grade, OS (18), and DFS time (17, 18). Conversely, others failed to find statistically significant correlations between clinicopathological parameters (7, 13, 14), including histological grade, stage (11, 17) and survival (13). In the present study, a correlation was observed only between c-erb-B2 multiplicity (P = 0.041). EGFR (c-erb-B1/her1) is a transmembrane tyrosine kinase with roles in proliferation, cell motility, and apoptosis. Increased expression and/or aberrant function of EGFR are associated with tumor progression and poor prognosis in many epithelial neoplasms, including HCC (7, 12, 13, 16). Although EGFR expression has been observed in 40-85% of HCCs in previous studies (7, 12, 13, 16, 44, 45), Nakopoulou et al. (11) reported EGFR-positive staining in only 3 out of 71 cases (4.2%). It was determined that 40% of the cases described here showed positive EGFR membrane staining, which is similar to most published studies (7, 12, 13, 16, 44, 45). There is considerable disparity in the literature regarding the relationship between EGFR expression and clinicopathologic features of HCC. EGFR has been correlated with the proliferation activity, stage, ALI, carcinoma differentiation (7), invasiveness, and recurrence (12, 44) and it is proposed to play an important role in carcinogenesis and HCC progression (7, 12, 16, 17, 46). However, some studies suggest that neither c-erb-B-2-oncoprotein nor EGFR are predominantly involved in the transformation of hepatocytes to a malignant phenotype and that their levels do not correlate with any of the main clinicopathologic features or survival (11, 13, 45). Here, EGFR was found to be correlated with stage, ALI, and AFP levels. Collectively, the findings suggest that EGFR remains an important potential therapeutic target for HCC, but further studies are necessary to demonstrate the exact impact of EGFR overexpression. PTEN, PI3K, and mTOR are known to be involved in carcinogenesis. PTEN, a tumor suppressor gene, is a negative regulator of the PI3K-Akt signaling pathway that promotes carcinogenesis. PTEN is mutated and inactivated at a high frequency in several cancers. Its loss leads to AKT kinase activation, which contributes to cell survival, growth, proliferation, and invasion (19, 23, 24). Hu et al. (20) and Wu et al. (21) demonstrated that reduced PTEN expression is involved in HCC pathogenesis and correlated with increased tumor grade, advanced disease stage, intrahepatic vascular embolism, and poor prognosis. Immunohistochemically, PTEN loss is correlated with mTOR overexpression (22). Villanueva et al. (24) found that aberrant mTOR signaling was present in 50% of HCC cases. Overexpression of mTOR is reportedly associated with tumor grade, ALI, TNM stage, high Ki-67 labeling, and other poor prognostic features in HCC (22, 23). mTOR expression was found in 22 cases (44%), but no significant relationship was observed between mTOR and PTEN. Mutations in PTEN and PI3K mutations are rare events (24). No immunohistochemical studies regarding PI3K expression in HCC were found. So, although it is not possible to make comparisons with previous publications, PI3K expression was observed in all assessed cases, but significant relationships with clinicopathological parameters were not found. To date, many studies have reported genetic and epigenetic alterations of cell-cycle regulatory proteins in HCC. P27 is a cyclin-dependent kinase inhibitor (CDKI) and negatively regulates cell cycle progression through the G1 phase; its expression is highest at G0/G1 and lowest during the S phase. P27 is a potent tumor suppressor in several human cancers, including HCC. Reduced/absent p27 nuclear expression is associated with poor prognosis in several carcinomas, including resistant phenotypes (25).

Some studies have reported significantly reduced p27 in HCC tissues compared to cirrhotic, HCV-infected, or normal liver tissue (27, 28, 30-32). Conversely, Tretiakova et al. (32) suggested that increased p27 expression was associated with higher tumor grade, supporting its role in cell cycle regulation in carcinogenesis and progression. Huang et al. (26) also reported higher p27 expression in tumor tissues compared to adjacent healthy tissue. In the current study, only 8% of cases were positive for p27. Many studies have reported that decreased p27 expression is a risk factor in HCC (25, 26, 28, 29, 32) and is significantly correlated with portal invasion, tumor invasiveness, advanced clinical stage, poor cellular differentiation, larger size, and intrahepatic and distant tumor metastasis (25, 28, 32). However, some reports found no relationships between p27 expression and tumor size, metastasis, AFP level, or ALI (26, 32). p27 positivity was observed only in 4 cases and correlations was found with AFP level (P = 0.047), multiplicity (P < 0.001), and c-erb-B2 expression (P = 0.011). Some authors have reported that p27 is an independent prognostic marker for DFS; poorer outcomes have been observed in cases with decreased p27 (28, 29). P27 has also been suggested to be an independent predictor of HCC recurrence (28). In the current study, p27 expression was inversely correlated with DFS and OS (P = 0.004 and P = 0.002, respectively). Although the current study findings seem to disagree with previous studies (28, 29), they may indirectly confirm the results of Tretiakova et al. (32), who reported that increased p27 expression was correlated with higher tumor grade. ERCC1 is a key DNA repair enzyme (33, 34). Fautrel et al. (33) reported increased ERCC1 expression associated with liver fibrogenesis and cancer. Ueda et al.,(34) found larger tumor size in a low expression group compared to a high expression group. According to some studies, increased ERCC1 expression could be associated with resistance to cisplatin-based therapy in HCC, similar to what is observed in non-small cell lung carcinoma (33, 34, 47). Therefore, IHC analysis for resected HCC tissues may be a useful predictor for the effectiveness of adjuvant chemotherapy (34). In contrast to these studies, Turhal et al. (35) suggested that ERCC1 expression could not be the only cause of cisplatin resistance because they only found ERCC1 expression in less than 2% (1/61 patients), whereas 90% of patients with HCC were resistant to cisplatin. Obviously there were many other factors involved in cisplatin resistance, such as the expression of multidrug resistance-associated proteins (35). ERCC1 expression was assessed in 26 HCC cases. Three cases were completely negative, and 16 cases with positive nuclei (61.5%) had H-scores < 1. In conclusion, c-erb-B2, EGFR, mTOR, and ERCC1 overexpression and p27 loss may play roles in hepatocarcinogenesis and may be significant predictors of aggressive tumor behavior. They were found to be correlated with some clinicopathologic features, therapy modalities, and survival time. These findings may help in planning new targeted treatment strategies for HCC; however, large scale in vitro and in vivo studies are needed.
